# The Effectiveness of Neuromuscular Warmups for Lower Extremity Injury Prevention in Basketball: A Systematic Review

**DOI:** 10.1186/s40798-021-00355-1

**Published:** 2021-09-16

**Authors:** Anna C. Davis, Nicholas P. Emptage, Dana Pounds, Donna Woo, Robert Sallis, Manuel G. Romero, Adam L. Sharp

**Affiliations:** 1 Center for Effectiveness and Safety Research, Kaiser Permanente, 100 South Los Robles Ave., Pasadena, CA 91101 USA; 2 Kaiser Permanente Southern California Department of Research and Evaluation, 100 South Los Robles Ave., Pasadena, CA 91101 USA; 3 Fontana Medical Center, Kaiser Permanente Southern California, 9961 Sierra Ave, Fontana, CA 92335 USA; 4 University of the Pacific, 3601 Pacific Ave., Stockton, CA 95211 USA; 5 Kaiser Permanente Bernard J Tyson School of Medicine, 98 South Los Robles Ave., Pasadena, CA 91101 USA

**Keywords:** Knee injury, Ankle injury, Evidence review, Balance, Strength, Agility, Training, Dynamic warmup

## Abstract

**Background:**

Neuromuscular warmups have gained increasing attention as a means of preventing sports-related injuries, but data on effectiveness in basketball are sparse. The objective of this systematic review was to evaluate evidence of the effectiveness of neuromuscular warmup-based strategies for preventing lower extremity injuries among basketball athletes.

**Methods:**

PubMed and Cochrane Library databases were searched in February 2019. Studies were included if they were English-language randomized controlled, non-randomized comparative, or prospective cohort trials, tested neuromuscular and/or balance-focused warmup interventions among basketball players, and assessed at least one type of lower extremity injury as a primary outcome. Criteria developed by the USPSTF were used to appraise study quality, and GRADE was used to appraise the body of evidence for each outcome. Due to heterogeneity in the included studies, meta-analyses could not be performed.

**Results:**

In total, 825 titles and abstracts were identified. Of the 13 studies which met inclusion criteria for this review, five were balance interventions (3 randomized controlled trials) and eight were multicomponent interventions involving multiple categories of dynamic neuromuscular warmup (5 randomized controlled trials). Authors of four of the studies were contacted to obtain outcome data specific to basketball athletes. Basketball specific results from the studies suggest significant protective effects for the following lower extremity injuries: ankle injuries (significant in 4 out of the 9 studies that assessed this outcome); ACL injuries (2 of 4 studies); knee injuries generally (1 of 5 studies); and overall lower extremity injuries (5 of 7 studies). All but one of the non-significant results were directionally favorable. Evidence was moderate for the effect of multicomponent interventions on lower extremity injuries generally. For all other outcomes, and for balance-based interventions, the quality of evidence was rated as low.

**Conclusion:**

Overall, the evidence is supportive of neuromuscular warmups for lower extremity injury prevention among basketball players. However, most studies are underpowered, some used lower-quality research study designs, and outcome and exposure definitions varied. Due to the nature of the study designs, effects could not be attributed to specific intervention components. More research is needed to identify the most effective bundle of warmup activities.

**Supplementary Information:**

The online version contains supplementary material available at 10.1186/s40798-021-00355-1.

## Key Points

- Available evidence supports the effectiveness of neuromuscular warmups for prevention of lower extremity injuries in basketball.
- Poor adherence to warmups and study design flaws impact the strength of the evidence.
- More research is needed to identify the necessary and sufficient components of basketball warmup routines.

## Background

Efforts to prevent sports-related injuries are widespread, and include many protective measures ranging from rules of play and protective equipment (e.g., helmets, padding, braces) to training and warmup activities (which may include a wide range of pre-play routines).

Static stretching—which has long been a key facet of traditional warmups in many sports [1]—can lead to improvements in range of motion and have other performance benefits [2]. However, static stretching has not been shown to decrease injuries when completed on its own, and as a result it is increasingly recommended that warmup activities include “dynamic” components [2–7]. Dynamic, or “neuromuscular” warmup activities can be defined as neuromuscular training programs that incorporate general (e.g., fundamental movements) and specific (e.g., sport-specific movements) strength and conditioning activities such as resistance, dynamic stability, balance, core strength, plyometric, and agility exercises [8, 9].

A growing body of evidence supports the effectiveness of dynamic warmup activities before play for protection against injury across a range of sports and player populations [6, 10–13]. Age-adjusted injury rates attributed to basketball in the USA are higher than for any other specific sport (3.3 per 1,000 persons overall) [14] According to a recent review, the majority of sports injuries in basketball (63.7%) are in the lower limbs [15]. However, the evidence regarding effectiveness of neuromuscular warmup activities for injury prevention among basketball athletes remains sparse.

Despite some preliminary reports about general injury prevention strategies for basketball [16], details about the interventions and information specific to warmup activities are lacking [10]. We conducted a systematic review in accordance with the methodology described by the Preferred Reporting Items for Systematic Reviews and Meta-Analyses (PRISMA) guidelines [17] to evaluate the evidence supporting the effectiveness of neuromuscular dynamic warmup activities (versus no warmup activities or “usual” warmup routines) for preventing lower extremity injuries among basketball athletes.

## Methods

### Search Strategy

The PubMed and Cochrane Library databases were searched on February 28, 2019 to identify primary research evaluating the effectiveness of warmup activities that include dynamic/neuromuscular movements for preventing lower extremity injuries in basketball players. The search terms were adapted from Taylor et al. 2015: “basketball[tiab] AND (warm[tiab] OR neuromuscular[tiab] OR stretch[tiab] OR strength[tiab] OR balance[tiab] OR agil*[tiab] OR land[tiab] OR “low extremity”[tiab] OR “lower extremity”[tiab] OR “low-extremity”[tiab] OR “lower-extremity”[tiab])” [16]. No date or language limits or other filters were applied to the searches.

### Study Inclusion and Exclusion Criteria

Peer-reviewed English-language randomized controlled trials, non-randomized comparative trials, and pre-post prospective cohort studies were eligible for inclusion; retrospective studies were excluded. Studies were included if they assessed at least one of the following as an outcome: occurrence of at least one type of lower-extremity injury; games, practices or time lost due to lower-extremity injury; or lower-extremity-injury-related withdrawal from sports participation. Studies that did not investigate at least one of these outcomes were excluded. Studies were considered if they included a neuromuscular/dynamic or balance focused warmup activity, including strength training, balance training, agility, and jumping/landing exercises. Studies that evaluated warmup activities combining both static stretching and dynamic exercises were included. Studies evaluating static stretching alone were excluded, as were those focusing exclusively on other types of interventions such as external knee and ankle supportive devices. Finally, studies were included if they were basketball specific or if basketball-specific data could be abstracted or obtained from the study authors; all data reported in this review are for the basketball participants in the included studies.

After the removal of duplicate results across the PubMed and Cochrane literature searches, a single author (NE) reviewed the titles and abstracts and removed papers not meeting eligibility criteria. A detailed full-text review of the remaining papers was performed to identify the final set of articles meeting all criteria. The same author examined the reference lists of the included studies, as well as literature review articles and meta-analyses identified during the literature searches, to identify any additional studies that might have been missed by the search strategy.

### Study Quality Assessment

One author (NE) critically appraised the individual studies to assess whether there were any significant methodological flaws or other limitations that could invalidate any of the published conclusions. Criteria adapted from those used by the U.S. Preventive Services Task Force (USPSTF) (see Additional file 1: Table A1) guided this assessment [18]. Each study was assigned a final rating of good, fair, or poor quality. Only studies without a control group were graded as poor quality. Comparative studies without random assignment to intervention or control conditions were graded as fair quality, as were randomized studies with methodological problems likely to introduce bias into the results (e.g., important and statistically significant baseline differences between groups, differential attrition between groups).

### Data Extraction

Detailed information was abstracted from the included studies by four reviewers (NE, DW, DP, AD), including study design, descriptive characteristics of the population (i.e., participant age, sex, and competitive level), the numbers of basketball athletes in the intervention and control groups, the proportion completing the study, and the numbers of ankle, knee and all lower-extremity injuries occurring in each group during the study. In order to obtain complete basketball-specific data from studies that incorporated multiple sports, we contacted the authors of four of the included studies [19–22] by email; all four provided data on numbers and types of injuries, athletes, and athlete-exposures for basketball athletes only, for the purpose of this review.

Other data collected from each study included the characteristics of the warmup interventions, including the program’s name, its specific components (i.e., static or dynamic stretches, jumping or plyometrics, strength training, balance or stability exercises, running, or agility training) and the individual exercises therein, length, duration and frequency of the intervention, whether the intervention included non-warmup components or special equipment, and information on the delivery of the intervention.

### Data Analysis

In this review, effectiveness results are presented for ankle injuries, ACL injuries, other knee injuries, and lower-extremity injuries generally. For studies that included basketball-specific results, we report their original findings (which were frequently the result of multivariate analyses that adjusted for possible confounders). For those studies in which the published results covered multiple sports, we used only their basketball-specific data (obtained from study authors or supplemental materials) to calculate risk ratios and their 95% confidence intervals comparing injury risks for basketball athletes in the intervention versus control group (or pre- and post-periods for studies without a comparison group); these calculations could not control for confounding factors.

Risk ratios (as well as odds ratios and hazard ratios) with a value of 1 indicate no risk difference between groups, while ratios < 1 suggest a reduced risk in the intervention group, and ratios > 1 an increased risk in the intervention group; a confidence interval on any of these measures that contains 1 indicates that the difference is not statistically significant. For example, a risk ratio of 1.25 can be interpreted as a risk in the intervention group that is 1.25 times higher than in the control group, all else being equal [23, 24]. Because specific outcome measures and methods of accounting for exposure time varied amongst the studies, this review focuses on statistical significance of the outcomes rather than magnitude of the point estimates.

Due to heterogeneity in the included studies with respect to study design, duration of follow-up, data reporting, and intervention characteristics, meta-analyses were not possible. As such, the presentation and discussion of the results of this review are qualitative in nature and focus on patterns of effect sizes and effect directions across the included trials. Statistical significance was defined at *p* = 0.05 and assessed using 95% confidence intervals or p-values, depending on the statistic.

Finally, the overall quality of the evidence for each outcome was assessed by one author (NE) using the Grading of Recommendations, Assessment, Development and Evaluation (GRADE) framework [25]. In GRADE, the quality of a body of evidence for a specific outcome is characterized as high, moderate, low, or very low after a consideration of the risk of bias, inconsistency, indirectness, imprecision, and publication bias. As noted by the creators of the GRADE system, the evidence rating is a subjective assessment.

## Results

### Review Statistics

**Fig. 1:**
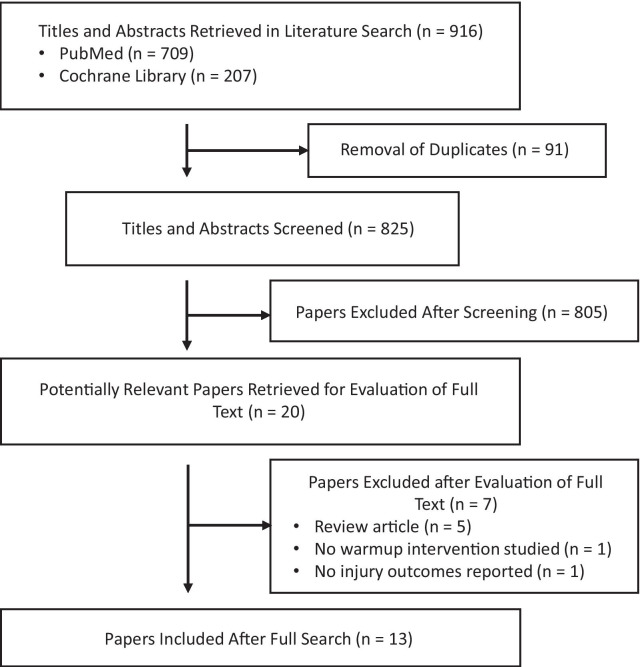
Flow diagram of studies identified, screened, and included through the literature search

Electronic searches yielded 916 results, including 825 unique papers. After screening the titles and abstracts of the full set of articles, the full texts of 20 papers were reviewed; of these 13 were included in this review [19–22, 26–34] and seven were excluded as shown in Fig. 1.

### Quality of the Included Studies

The 13 studies in the final sample included eight cluster randomized controlled trials (RCTs) [20, 21, 26–30, 32], three non-randomized comparative studies [19, 31, 34], and two prospective cohort studies [22, 33]. Quality review using USPSTF criteria rated five of the cluster RCTs as good quality [21, 26–29], three cluster RCTs and the three non-randomized comparative studies as fair quality [19, 20, 30–32, 34], and the two cohort studies as poor quality[22, 33] (Table 1). The studies varied widely with respect to consistently reporting the items covered by the quality assessment. Detailed elements of the USPSTF quality assessment are contained in Additional file 1: Table A1).

**Table 1:** Study designs, USPSTF quality rating, analysis methods, and outcome measures assessed

Study	Study design	USPSTF quality rating	Analysis method	Outcome measures assessed			
				All lower-extremity injuries	Ankle injuries	ACL injuries	Knee injuries (general)
*Balance interventions*							
McGuine et al. [21]	Cluster RCT	Good quality	Multivariate Cox proportional hazards model of incidence rates		X		
Cumps et al. [19]	Non-randomized controlled trial	Fair quality	T-test of relative risk		X		
Emery et al. [29]	Cluster RCT	Good quality	Multivariate Poisson regression of relative risk	X	X		
Eils et al. [28]	Cluster RCT	Good quality	Logistic regression of odds ratio		X		
Riva et al. [22]	Prospective cohort	Poor quality	ANOVA of incidence rates	X^a^	X		X
*Multicomponent interventions*							
Hewett et al. [31]	Non-randomized controlled trial	Fair quality	χ^2^ of incidence rates			X	
Pfeiffer et al. [34]	Non-randomized controlled trial	Fair quality	Exact test of odds ratio			X^a^	
LaBella et al. [20]	Cluster RCT	Fair quality	Exact test of incidence rates	X^a^	X^a^		X^a^
Longo et al. [32]	Cluster RCT	Fair quality	Exact test of odds ratio	X	X		X
Aerts et al. [26]	Cluster RCT	Good quality	Multilevel Cox proportional hazards model of hazard ratio	X			
Bonato et al. [27]	Cluster RCT	Good quality	χ^2^ of incidence rates	X^a^	X	X	X
Foss et al. [30]	Cluster RCT	Fair quality	Mixed-model comparison of incidence rates	X^a^	X		X
Omi et al. [33]	Prospective Cohort	Poor quality	*χ*^2^ of relative risk			X	

X: reported in original study

X^a^: not reported in original study, but a basketball-specific risk ratio could be computed based on basketball-specific numbers of injuries and athletes

As shown in Table 1, each outcome measure could be reported for only select studies from the total of 13. Knee injury outcomes (either ACL or general knee injuries) could be reported for eight of the studies [20, 22, 27, 30–34], and ankle injury outcomes for nine of the studies [19–22, 27–30, 32]. Three of the studies reported on lower-extremity injuries in general [26, 29, 32]. We were able to calculate a combined general lower-extremity injury outcome by adding together data on knee and ankle injuries for an additional four studies [20, 22, 27, 30]. The specific outcome definitions in the studies varied, such as focusing only on non-contact injuries. Studies reported injury incidence per player-hours [19, 26, 27, 29, 32] or player-exposures [20–22, 28, 30, 31, 33, 34] (usually defined as one athlete participating in one game or practice).

A description of the study participants, comparison condition, and a high-level summary of the warmup intervention for each study is provided in Table 2. A more detailed tabulation of the warmup activities in each of the included studies is provided in Additional file 1: Table A2.

**Table 2:** Summary of included studies and intervention components

Study	Basketball population	Age group	Elite (Y/N)	Sex	N intervention—basketball	N control—basketball	Warmup intervention components					Comparison	Time horizon
							Stretching—static	Dynamic warmup	Jumping/plyometrics	Strength	Balance/ stability		
*Balance interventions*													
McGuine et al. [21]	High-school basketball players	Youth	N	M/F	122	113					X	Normal warmup, training and/or conditioning practices	1 season
Cumps et al. [19]	Elite youth and young-senior basketball players	Youth & Adult	Y	M/F	29	25					X	Normal warmup, training and/or conditioning practices	22 weeks
Emery et al. [29]	High-school competitive basketball players	Youth	N	M/F	494	426					X	Standardized basic warmup taught to both intervention and control groups	1 year
Eils et al. [28]	German professional basketball players	Adult	Y	M/F	96	102					X	Normal warmup, training and/or conditioning practices	1 season
Riva et al. [22]	Professional Italian basketball players	Adult	Y	NR	24	24					X	NA—comparison is pre-period	6 years
*Multicomponent interventions*													
Hewett et al. [31]	High-school competitive female basketball players	Youth	N	F	84	189	X	X	X	X		Normal warmup, training and/or conditioning practices	1 season
Pfeiffer et al. [34]	High-school competitive female basketball players	Youth	N	F	191	319		X	X			Normal warmup, training and/or conditioning practices	2 seasons
LaBella et al. [20]	High-school competitive female basketball players	Youth	N	F	236	161		X	X	X		Normal warmup, training and/or conditioning practices	1 season
Longo et al. [32]	Elite precollegiate male basketball players	Youth & Young Adult	Y	M	80	41		X	X	X	X	Normal warmup, training and/or conditioning practices	1 season
Aerts et al. [26]	Competitive adult basketball players	Youth & Adult	N	M/F	129	114			X	X		Normal warmup, training and/or conditioning practices	6 months
Bonato et al. [27]	Professional Italian female basketball players	Adult	Y	F	86	74		X	X	X		Normal warmup, training and/or conditioning practices	1 season
Foss et al. [30]	Middle-school and high-school female basketball players	Youth	N	F	126	121			X	X		Sham protocol with resisted running using elastic bands	1 season
Omi et al. [33]	Collegiate female basketball players	College	N	F	448	309			X	X	X	NA—comparison is pre-period	8 years

The warmup activities ranged in duration from 5 to 90 minutes; the majority were designed to be 25 minutes or less, completed 2–4 times per week (Table 2 and Additional file 1: Table A2). The interventions contained a wide variety of exercises and activities (Table 2 and Additional file 1: Table A2). We categorized the warmup activities into five domains: Static Stretching; Dynamic/Neuromuscular; Jumping/Plyometrics; Strength; and Balance/Stability. Among the 13 included studies, five studies focused specifically on balance/stability interventions [19, 21, 22, 28, 29]. The remaining eight studies tested a warmup intervention that incorporated activities from multiple domains; for brevity, these are called “multicomponent” warmup interventions throughout the remainder of this review [20, 26, 27, 30–34]. Because of the nature of the original study designs, it was not possible to evaluate the effectiveness of the distinct components of the multicomponent warmup interventions separately from one another.

The interventions in six of the 13 studies were tested among athletes of high-school age or younger [20, 21, 29–31, 34], while the remainder were tested in collegiate and/or adult populations including professional or elite athletes (Table 2). The intervention groups ranged in size from 24 to 494 athletes, and comparison groups were often equivalent (Table 2).

### Evidence of Effectiveness

#### All Lower-Extremity Injuries

A general outcome for all lower extremity injuries was available for seven studies (Table 3); three of these reported the estimate directly [26, 29, 32] and we computed a combined knee and ankle injury outcome for the other four [20, 22, 27, 30]. Five of the seven studies tested multicomponent interventions, while two were balance-only interventions. The risk of lower extremity injuries was significantly lower among intervention group athletes in one of the balance-based interventions [22]. Among the five multicomponent interventions, four had a statistically significant protective effect for general lower extremity injuries [20, 26, 27, 32]. Based on the GRADE assessment, the evidence for the effectiveness of multicomponent interventions on lower extremity injuries generally is of moderate quality, with the only area of concern being the baseline non-equivalence of the intervention and control groups in two studies. The quality of the evidence for balance-based interventions was rated as low, because of the risk of bias and imprecision arising from small numbers of outcome events. The components of the GRADE ratings are summarized in Additional file 1: Tables A3 and A4.

**Table 3:** Effectiveness—all lower extremity injuries

Study	Intervention group		Control group		Point estimate	Confidence measure (95% confidence interval or *p* value)
	Injuries	*N*	Injuries	*N*		
*Balance interventions*						
McGuine et al. [21]	–	–	–	–	–	–
Cumps et al. [19]	–	–	–	–	–	–
Emery et al. [29]	106	494	111	426	RR = 0.83	0.57–1.19
Eils et al. [28]	–	–	–	–	–	–
Riva et al. [22]	8	24	33	24	RR = 0.21	0.10–0.45*
*Multicomponent interventions*						
Hewett et al. [31]	–	–	–	–	–	–
Pfeiffer et al. [34]	–	–	–	–	–	–
LaBella et al. [20]	26	236	48	161	RR = 0.37	0.24–0.57*
Longo et al. [32]	10	80	11	41	OR = 0.40	0.194–0.84*
Aerts et al. [26]	18	129	28	114	HR = 0.40	0.16–0.99*
Bonato et al. [27]	11	86	45	74	RR = 0.25	0.14–0.45*
Foss et al. [30]	47	126	53	121	RR = 0.85	0.63–1.15
Omi et al. [33]	–	–	–	–	–	–

The numerator in all injury rates (reported and computed) is the number of total injuries, not the number of persons injured

Emery et al. [29], LaBella et al. [20], Longo et al. [32], and Aerts et al. [26] reported data for All LEIs which they defined in various ways. For other studies, we computed an “All LEIs” outcome when possible, by adding together injury data as follows: Riva et al. [22], knee and ankle injuries; Bonato et al. [27], knee, ACL and ankle injuries; Foss et al. [30], knee and ankle injuries

“ARR” is adjusted risk ratio; “HR” is hazard radio; “NR” is not reported; “OR” is odds ratio; “RR” is risk ratio

“*” = statistically significant at *p* = 0.05 level

#### Ankle Injuries

Nine studies examined the effect of warmup activities on ankle injuries specifically (Table 4) [19–22, 27–30, 32]. Five of these studies tested balance/stability only interventions, while the other four tested multicomponent interventions. Among these nine studies, the results for four of the balance-based interventions showed a statistically significant reduction in risk of ankle injuries [19, 21, 22, 28]. The results from the remaining five studies were not statistically significant for this outcome. Based on the GRADE assessment, the evidence for the effectiveness of warmup interventions on ankle injuries was rated as low, because of the risk of bias and imprecision arising from small numbers of outcome events. The components of the GRADE ratings are summarized in Additional file 1: Tables A3 and A4.

**Table 4:** Effectiveness—ankle Injuries

Study	Intervention group		Control group		Point estimate	Confidence measure (95% confidence interval or *p* value)
	Injuries	*N*	Injuries	*N*		
*Balance interventions*						
McGuine et al. [21]^a^	10	122	15	113	RR = 0.56	0.33–0.95*
Cumps et al. [19]^b^	5	26	9	24	RR = 0.30	0.11–0.84*
Emery et al. [29]	62	494	76	426	RR = 0.71	0.45–1.13
Eils et al. [28]^b^	7	89	21	99	OR = 0.36	0.15–0.84*
Riva et al. [22]	6	24	28	24	RR = 0.19	0.08–0.46*
*Multicomponent interventions*						
Hewett et al. [31]	–	–	–	–	–	–
Pfeiffer et al. [34]	–	–	–	–	–	–
LaBella et al. [20]	16	236	18	161	RR = 0.61	0.32–1.15
Longo et al. [32]	3	80	2	41	OR = 0.79	0.21–3.04
Aerts et al. [26]	–	–	–	–	–	–
Bonato et al. [27]	9	86	26	74	*χ*^2^ test	*p* = 0.51
Foss et al. [30]	12	126	17	121	ARR = 1.65	0.78–5.57
Omi et al. [33]	–	–	–	–	–	–

The numerator in all injury rates (reported and computed) is the number of total injuries, not the number of persons injured

“ARR” is adjusted risk ratio; “HR” is hazard radio; “NR” is not reported; “OR” is odds ratio; “RR” is risk ratio

“*” = statistically significant at *p* = 0.05 level

^a^Estimates reported by McGuine et al. [21] include both soccer and basketball players. A separate RR for basketball players was not included, but a multivariate analysis suggested that the effectiveness of the intervention did not differ significantly by sport

^b^Results shown from Cumps et al. [19] and Eils et al. [28] are ‘as treated’ estimates

#### ACL Injuries

A total of four studies examined the effect of warmup activities on ACL injuries; all of them tested multicomponent interventions (Table 5) [27, 31, 33, 34]. None of the studies focusing exclusively on balance/stability interventions provided results specifically on ACL injuries. Results from two of the four studies were statistically significant, suggesting a protective effect based on the intervention [27, 33]. Based on the GRADE assessment, the evidence for the effectiveness of warmup interventions on ACL injuries was rated as low, because of the risk of bias and imprecision arising from small numbers of outcome events. The components of the GRADE ratings are summarized in Additional file 1: Tables A3 and A4.

**Table 5:** Effectiveness—knee injuries (ACL)

Study	Intervention group		Control group		Point estimate	Confidence measure (95% confidence interval or *p* value)
	Injuries	*N*	Injuries	*N*		
*Balance interventions*						
McGuine et al. [21]	–	–	–	–	–	–
Cumps et al. [19]	–	–	–	–	–	–
Emery et al. [29]	–	–	–	–	–	–
Eils et al. [28]	–	–	–	–	–	–
Riva et al. [22]	–	–	–	–	–	–
*Multicomponent interventions*						
Hewett et al. [31]^a^	2	84	5	189	*χ*^2^ test	*p* = 0.89
Pfeiffer et al. [34]^b^	3	191	2	319	RR = 4.29	0.72–25.7
LaBella et al. [20]	–	–	–	–	–	–
Longo et al. [32]	–	–	–	–	–	–
Aerts et al. [26]	–	–	–	–	–	–
Bonato et al. [27]	0	86	7	74	*χ*^2^ test	*p* = 0.04*
Foss et al. [30]	–	–	–	–	–	–
Omi et al. [33]	9	448	16	309	RR = 0.38	**0.17**–0.87*

The numerator in all injury rates (reported and computed) is the number of total injuries, not the number of persons injured

“RR” is risk ratio; “OR” is odds ratio; “HR” is hazard radio; “ARR” is adjusted risk ratio; “NR” is not reported

“*” = statistically significant at *p* = 0.05 level

^a^Hewett et al. [31] data are for ACL and MCL injuries among female athletes only

^b^Pfeiffer et al. [34] reported non-contact ACL injuries

#### Knee Injuries

Five studies examined the effect of warmup activities on a general outcome for knee injuries, including ACL, MCL, and other knee injuries (Table 6) [20, 22, 27, 30, 32]. Four of these studies tested a multicomponent intervention, while one was a balance intervention. One of the five studies that evaluated knee injuries demonstrated a statistically significant protective effect based on the intervention [27]. Based on the GRADE assessment, the evidence for the effectiveness of warmup interventions on knee injuries was rated as low, because of the risk of bias and imprecision arising from small numbers of outcome events. The components of the GRADE ratings are summarized in Additional file 1: Tables A3 and A4.

**Table 6:** Effectiveness—knee injuries (general)

Study	Intervention group		Control group		Point estimate	Confidence measure (95% confidence interval or *p* value)
	Injuries	*N*	Injuries	*N*		
*Balance interventions*						
McGuine et al. [21]	–	–	–	–	–	–
Cumps et al. [19]	–	–	–	–	–	–
Emery et al. [29]	–	–	–	–	–	–
Eils et al. [28]	–	–	–	–	–	–
Riva et al. [22]	2	24	5	24	RR = 0.36	0.07–1.83
*Multicomponent interventions*						
Hewett et al. [31]	–	–	–	–	–	–
Pfeiffer et al. [34]	–	–	–	–	–	–
LaBella et al. [20]^a^	7	236	9	161	RR = 0.53	0.20–1.40
Longo et al. [32]	5	80	2	41	OR = 1.21	0.358–4.11
Aerts et al. [26]	–	–	–	–	–	–
Bonato et al. [27]	2	86	12	74	*χ*^2^ test	***p*** = 0.037*
Foss et al. [30]	35	126	36	121	ARR = 1.07	0.72–1.65
Omi et al. [33]	–	–	–	–	–	–

The numerator in all injury rates (reported and computed) is the number of total injuries, not the number of persons injured

“RR” is risk ratio; “OR” is odds ratio; “HR” is hazard radio; “ARR” is adjusted risk ratio; “NR” is not reported

“*” = statistically significant at *p* = 0.05 level

^a^LaBella et al. [20] reported non-contact knee injuries

### Discussion

Our review of the literature evaluated the current state of the evidence to support the effectiveness of warmup activities for lower extremity injury prevention in basketball. We expanded upon earlier reports by describing details about the study populations, intervention components and time horizon of evaluation [16]. Detailed descriptions of the warmup interventions are available in the Additional file 1: Table A2.

Overall, 13 studies including eight cluster RCTs, three comparative non-randomized studies, and two prospective cohort studies were identified. Five of these tested balance-focused interventions, while the remaining eight tested multicomponent interventions including a mix of strength training, balance training, agility/dynamic warmup, and jumping/landing exercises. We collected information on ankle injuries (available for 9 of the 13 studies), ACL injuries (4 studies), knee injuries generally (5 studies), and overall lower extremity injuries (7 studies). Though the included studies varied in many ways, most found significant decreases in general lower extremity injuries for basketball players (5 out of 7 studies). Fewer studies found warmups prevented ankle injuries specifically (4 of 9); all of these were balance/stability interventions. Only one (out of 5) multicomponent warmup intervention decreased knee injuries, and two (out of 4) multicomponent warmup interventions decreased ACL injuries specifically.

While effect measures varied among the studies and some effect sizes were small, we believe that any reduction in injuries is meaningful to the athlete and should be viewed positively. This is particularly true for ACL injury, where downstream costs and consequences can be very significant. Furthermore, warmup activity-based injury prevention programs are generally low risk and have minimal costs when not requiring specialized equipment, which may be an important factor in adoption and adherence [6].

Overall, this systematic review supports previous reports from other sports which have found that neuromuscular warmup routines can decrease lower extremity injuries [6, 13, 35–37]. Possible physiologic mechanisms include stimulating joint position sense and kinesthesia during dynamic/neuromuscular warmups, to prime the body for specific movement patterns of the respective sport through multi-joint and multiplanar repetitions. However, the evidence for warmup effectiveness in injury prevention is not as consistent as one might hope, and the largest study of good quality in our review did not report a statistically significant result for basketball athletes on either ankle or all lower extremity injuries [29]. This may be explained in part by low compliance with the intervention: 60% of intervention group athletes in this study participated at all and the median number of sessions was 9 over six weeks. The intervention tested in this study was the use of a home wobble board training program (balance training), and the intervention and control groups were both taught a standardized warmup routine that was used as the comparison condition [29]; many other studies did not standardize the control condition.

Adherence to many of the interventions was low, even with elements of some study designs that likely supported intervention fidelity such as observation of training sessions by study staff [26]. This echoes results from another recent warmup trial in which intervention adherence was very poor [38]. It is possible that some of the warmup activities included in the studies in this review would have been effective with better adherence/implementation fidelity. Other studies have demonstrated a significant association between improved compliance with warmup interventions and decreased injury rates [13]. There is some literature regarding factors that either increase or decrease adherence/fidelity to warm-up routines [39–41]; however, assessing reasons for low adherence in the included studies was not in the scope of this review.

Many of the included studies suffered from methodological limitations, such as non-equivalence between groups at baseline, and non-randomized or non-comparative research designs. Moreover, many of these studies were small and few ankle or knee injuries occurred in the study populations, which limited the precision with which effects could be estimated. As such, the GRADE quality of the evidence for both knee and ankle injuries was rated as low, while the evidence for lower-extremity injuries in general was rated low for balance-based programs and moderate for multicomponent programs.

It is unclear which warmup activities specifically are most useful for the prevention of lower extremity injuries. In part this is due to the high variability in the structure of the warmup interventions, which were generally combinations of many discrete exercises completed in sequence, with varying duration, repetition, frequency, and intensity. It is therefore impossible to identify which specific warmup components may be associated with superior injury prevention based on the current evidence. In addition, authors sometimes used differing language for exercises that are the same or very similar with respect to mechanics and metabolic demand. For example, studies variously reported using ‘single leg lateral leaps,’ ‘lateral jump and hold,’ ‘bounding in place,’ and ‘side jump single leg,’ which are all mechanically similar. These inconsistencies make it difficult to compare the individual components of the complex interventions tested, and arguably are a barrier to implementing any of these interventions with fidelity.

Our review had limitations. We only searched the PubMed and Cochrane databases for manuscripts in English. The search was conducted in February 2019; however, an updated search conducted in July 2021 identified only one additional study [38] that met our inclusion criteria. This study of the FIFA 11 + injury prevention program in high school athletic teams reported a null finding for reduction of lower extremity injuries among basketball players and its inclusion therefore would not have substantially altered our conclusions. The studies in this review may have been underpowered to find an effect as most reported very few injury events, which can lead to insignificant findings. Furthermore, only five of the 13 studies in this review were rated as good quality based on their use of a strong comparator and other controls to limit threats to validity [21, 26–29]. Among the good quality studies alone, the evidence generally favored the effectiveness of warmup activities for decreasing injuries.

The included studies varied in their choice of primary outcome measure and their statistical analysis approach. The general lower-extremity injury outcome measure definition varied somewhat between included studies, such as studying non-contact injuries only [34], focusing only on sprain-type injuries [22], or including foot, thigh, hip and groin in the general outcome measure for lower extremity injury [32]. Another limitation was that intervention effectiveness could not be expressed using a hazard ratio outcome because information on exposure time was reported inconsistently across studies (e.g., hours of play vs. numbers of practices/games). Due to the heterogeneity of the included studies and their inconsistent measurement of exposure and reporting of outcomes data, it was not possible to pool results for meta-analysis.

In the absence of clear and unambiguous evidence about what warmup activities are effective, it is unlikely that many non-professional athletes consistently engage in warmup activities that will be optimally effective for injury prevention. This highlights the need to develop, disseminate and implement injury preventing warmups [42]. Moreover, previous studies have shown that initial benefits of a neuromuscular warmup routine decline if adherence wanes over time [43]. Thus, to be successful, a lower extremity injury prevention approach must be sustainable and used consistently. Once evidence accumulates about warmup activities that are effective for injury prevention under ideal trial circumstances, pragmatic studies are needed to build evidence about the implementation and dissemination of warmup activities in youth sports so that they can be sustained over time for maximal benefit; the limited evidence that exists suggests that there are many implementation challenges [38–40, 44, 45].

Additional research could address many of the limitations in the available studies. Careful randomization in the study design with intent to treat analyses could mitigate concerns about bias. Moreover, the small numbers of ankle and knee injuries suggest the need for larger trials. Greater standardization of the control conditions would increase the comparability of future studies. Finally, stepped-wedge trial designs could be used to identify which intervention components have greater effects on outcomes.

## Conclusions

Warmup activities are likely an effective strategy to reduce lower-extremity injuries in basketball players, although not all of the intervention strategies in this review demonstrated a protective effect. The best quality evidence (moderate) was for the effect of multicomponent interventions on lower extremity injuries generally. Specifically, four multicomponent interventions consisting of various exercises involving jumping/plyometrics, strength training and dynamic warmup activities were associated with a significantly lower risk of general lower extremity injuries among basketball players. However, given the nature of these warmup interventions (complex, multi-part routines) and the mixed quality of the study designs, it is unclear which components of warmup interventions are most effective in the prevention of lower extremity injury. For all other outcomes, and for balance-based interventions, the quality of evidence was low.

## Supplementary Information

**Additional file 1.** USPSTF and GRADE Quality Ratings and Detailed Inventories of the Warmup Interventions.

## Data Availability

Not applicable.

## Abbreviations

ACL: Anterior cruciate ligament
MCL: Medial collateral ligament
USPSTF: U.S. Preventive Services Task Force
GRADE: Grading of Recommendations, Assessment, Development and Evaluation
PRISMA: Preferred Reporting Items for Systematic Reviews and Meta-Analyses
RCT: Randomized controlled trial
RR: Risk ratio
OR: Odds ratio
HR: Hazard radio
ARR: Adjusted risk ratio
NR: Not reported
