# Supplementary material for: The Effectiveness of Neuromuscular Warmups for Lower Extremity Injury Prevention in Basketball: A Systematic Review
Source: Sports Med Open. 2021 Sep 16;7:67. doi: 10.1186/s40798-021-00355-1 (PMC8446130; doi:10.1186/s40798-021-00355-1)
Supplement: Supplementary file 1 — Additional file 1. USPSTF and GRADE Quality Ratings and Detailed Inventories of the Warmup Interventions. [file 40798_2021_355_MOESM1_ESM.pdf]

ADDITIONAL FILE 1

Table A1. USPSTF Quality Assessment of Included Studies

**Reference**	**Study design**	**Valid random assignment?**	**Allocation concealed?**	**Groups similar at baseline?**	**Eligibility criteria specified?**	**Measurements: equal, reliable, valid**	**Blinding of 1) outcomes assessors 2) providers 3) patients**	**Attrition and differential follow-up?**	**Handling of missing data**	**Adequate adherence to treatment?**	**Funding source**	**Other Notes**	**Overall quality**
**McGuine et al., 2006 (1)**	Cluster RCT	Yes	Yes	Yes	Yes	Likely	No	1% failed to complete the study, not reported by study group	Not described	91% compliance in intervention group	Non-profit		Good
**Cumps et al., 2007 (2)**	Non-randomized controlled trial	N/A	No	No (intervention group taller)	No	Uncertain	No	8% of intervention athletes, 6% of control athletes failed to complete the study	Subjects with missing data excluded	Not assessed.	Government		Fair
**Emery et al., 2007 (3)**	Cluster RCT	Yes	Yes	Yes	Yes	Likely	Yes for participants and providers, uncertain for outcomes assessors	2% of intervention athletes, 0% of control athletes failed to complete the study	Missing exposure data imputed as mean exposure hours by age and sex when entire team's data were missing, or as team mean exposure data when individual data	60% of intervention athletes complied with home-based component. Compliant athletes completed median 9 sessions over 6 weeks. No data provided on	Non-profit and government	Study recruitment did not reach threshold specified in power analysis	Good

Reference	Study design	Valid random assignment?	Allocation concealed?	Groups similar at baseline?	Eligibility criteria specified?	Measurements: equal, reliable, valid	Blinding of 1) outcomes assessors 2) providers 3) patients	Attrition and differential follow-up?	Handling of missing data	Adequate adherence to treatment?	Funding source	Other Notes	Overall quality
									missing. Other missing information not imputed	adherence to team warmup component.			
**Eils et al., 2010 (4)**	Cluster RCT	Yes	Yes	No (intervention subjects younger, more active)	Yes	Uncertain	Yes for participants, no for others	16% of intervention athletes, 11% of control subjects failed to complete the study	Not described	Not assessed	Government		Good
**Riva et al., 2016 (5)**	Prospective cohort study	N/A	N/A	N/A	No	Likely	No	Not reported	Not described	100% compliance	No external support		Poor
**Hewett et al., 1999 (6)**	Non-randomized controlled trial	N/A	No	Uncertain	No	Uncertain	No	6% of intervention athletes failed to complete the study	Not described	70% of intervention athletes completed the full program.	Non-profit		Fair
**Pfeiffer et al., 2006 (7)**	Non-randomized controlled trial	N/A	No	Uncertain	No	Uncertain	No	Not reported	Not described	Intervention athletes completed 18 sessions per week.	Non-profit		Fair
**LaBella et al., 2011 (8)**	Cluster RCT	Yes	Yes	No (racial/ethnic differences between groups, intervention subjects more likely to have prior strength/conditioning, play on multiple teams)	Yes	Uncertain	No	3% of intervention athletes, 5% of control athletes failed to complete the study	Not described	All 28 intervention-group coaches used some prescribed exercises, 70% used at least half of the prescribed exercises.	Non-profit		Fair
**Longo et al., 2012 (9)**	Cluster RCT	Yes	Yes	No (intervention subjects younger, shorter, lighter)	No	Uncertain	Yes for outcomes assessors, no for	0 athletes failed to complete the study	Not described	100% compliance in intervention group.	Not reported		Fair

Reference	Study design	Valid random assignment?	Allocation concealed?	Groups similar at baseline?	Eligibility criteria specified?	Measurements: equal, reliable, valid	Blinding of 1) outcomes assessors 2) providers 3) patients	Attrition and differential follow-up?	Handling of missing data	Adequate adherence to treatment?	Funding source	Other Notes	Overall quality
							participants and providers						
**Aerts et al., 2013 (10)**	Cluster RCT	Uncertain	Yes	Yes	Yes	Likely	Yes for participants, uncertain for others	30% of intervention athletes, 18% of control athletes failed to complete the study	Not described	66% of intervention teams performed the warmup at the recommended frequency. Overall session attendance by individual athletes was 60%.	Government		Good
**Bonato et al., 2018 (11)**	Cluster RCT	Yes	Yes	Yes	Yes	Likely	Yes for patients and outcomes assessors, no for providers	0 athletes failed to complete the study	Not described	78% compliance in the intervention group.	No external support		Good
**Foss et al., 2018 (12)**	Cluster RCT	Uncertain	Uncertain	Uncertain	No	Uncertain	Uncertain	Not reported	Not described	Not assessed	Government		Fair
**Omi et al., 2018 (13)**	Prospective cohort study	N/A	N/A	N/A	No	Uncertain	No	13% did not complete the first intervention period. 42% did not complete the 2nd intervention period.	Not described	Compliance ranged from 88% to 91%	Not reported		Poor

Table A2. Details of Neuromuscular Warmup Interventions Abstracted from the Original Included Studies

Study	Detailed Components of Warmup Interventions	Duration of sessions	Frequency of sessions	Duration of intervention period	Adaptation over time	Non-warmup components of the intervention and/or special equipment
**McGuine et al., 2006 (1)**	**Balance Training:**<br>Exercises performed 30 seconds per leg, alternating with 30-second rest between reps:<br>Single-leg stance (alone, while swinging raised leg or performing functional activities, static or while rotating board), single-leg squat, swinging raised leg, double-leg stance while rotating board	NR for preseason (weeks 1-4), 10 mins for weeks 5+	5 days/week in preseason, 3 days/week during season	4 weeks of preseason; season length NR	Stable intensity weeks 1-4, decreased intensity beginning week 5	Simple balance board.<br><br>Trainer monitored for compliance but no other components of the intervention.
**Cumps et al., 2007 (2)**	**Balance Training:**<br>Week 1: Basketball position: feet next to each other shoulder width on the ball of the feet without semi-globe (Session 1 = eyes open focused on ball / Session 2 = eyes open not focused on ball / Session 3 = eyes closed)<br>-stance: sweeps<br>-dribbling: push & pull dribble forward to backwards next to feet<br>-aberdeen: turn ball around head, trunk and legs in figure 8<br>-passing on the ball of the feet: hands up straight and diagonal passing<br><br>Week 2: Tandem stance: one foot behind the other on a straight line on the ball of the feet without semi-globe (Session 1 = eyes open focused on ball / Session 2 = eyes open not focused on ball / Session 3 = eyes closed)<br>-stance: sweeps<br>-dribbling: power dribble high and low and changing hand between the legs<br>-aberdeen: turn ball around head, trunk and legs in figure 8<br>-passing on the ball of the feet: hands up with 2 balls straight passing<br><br>Week 3: One leg stance without semi-globe (Session 1 = eyes open focused on ball / Session 2 = eyes open not focused on ball / Session 3 = eyes closed)<br>-stance: sweeps<br>-dribbling: push & pull dribble<br>-aberdeen: figure 8 between the legs<br>-passing on the ball of the feet: bad passing with tennis ball<br><br>Week 4: Jumping tasks with eyes open without semi-globe (Session 1 = 2 feet together / Session 2 = from one foot to the other / Session 3 = one foot)<br>-stance: mirror the teammate<br>-dribbling: push & pull dribble + jump into pass<br>-aberdeen: figure 8 between the legs + scissors jump<br>-passing on the ball of the feet: jump to bad pass with tennis ball<br><br>Week 5-8: Basketball position: feet next to each other shoulder width on the ball of the feet with semi-globe flat side down (Session 1 = eyes open focused on ball / Session 2 = eyes open not focused on ball / Session 3 = eyes closed)<br>-stance: stance / sweeps / sweeps + mirror the ball with eyes open / ball fight	5-10 mins per session	3 times per week	22 weeks	Increasing difficulty and safety of the balance skill; divided into 4 different phases determined by position of semi-globes and feet	Balance semi-globes

Study	Detailed Components of Warmup Interventions	Duration of sessions	Frequency of sessions	Duration of intervention period	Adaptation over time	Non-warmup components of the intervention and/or special equipment
	-dribbling: power dribble high & low / push & pull dribble / hockey dribble / L-dribble<br>-aberdeen: around trunk / figure 8 between the legs / head-trunk-figure 8 / scissors<br>-passing on the ball of the feet: chest pass/ hands up straight and diagonal passing / hands up with 2 balls straight passing / bad passing with tennis ball<br><br>Week 9-12: Tandem stance: one foot behind the other on a straight line on the ball of the feet with semi-globe flat side down (Session 1 = eyes open focused on ball / Session 2 = eyes open not focused on ball / Session 3 = eyes closed)<br>-stance: stance / sweeps / sweeps + mirror the ball with eyes open / ball fight<br>-dribbling: power dribble high & low / push & pull dribble / hockey dribble / L-dribble<br>-aberdeen: around trunk / figure 8 between the legs / head-trunk-figure 8 / scissors<br>-passing on the ball of the feet: chest pass/ hands up straight and diagonal passing / hands up with 2 balls straight passing / bad passing with tennis ball<br><br>Week 13-16: One leg stance with semi-globe flat side down (Session 1 = eyes open focused on ball / Session 2 = eyes open not focused on ball / Session 3 = eyes closed)<br>-stance: stance / sweeps / sweeps + mirror the ball with eyes open / ball fight<br>-dribbling: power dribble high & low / push & pull dribble / hockey dribble / L-dribble<br>-aberdeen: around trunk / figure 8 between the legs / head-trunk-figure 8 / scissors<br>-passing on the ball of the feet: chest pass/ hands up straight and diagonal passing / hands up with 2 balls straight passing / bad passing with tennis ball<br><br>Week 17-18: Basketball position: feet next to each other shoulder width on the ball of the feet with semi-globe side down (Session 1 = eyes open focused on ball / Session 2 = eyes open not focused on ball / Session 3 = eyes closed)<br>-stance: stance / sweeps<br>-dribbling: push & pull dribble / power dribble high & low<br>-aberdeen: around trunk / figure 8 between the legs<br>-passing on the ball of the feet: chest pass/ hands up straight and diagonal passing<br><br>Week 19-20: One leg stance with semi-globe side down (Session 1 = eyes open focused on ball / Session 2 = eyes open not focused on ball / Session 3 = eyes closed)<br>-stance: stance / sweeps<br>-dribbling: push & pull dribble / power dribble high & low<br>-aberdeen: around trunk / figure 8 between the legs<br>-passing on the ball of the feet: chest pass/ hands up straight and diagonal passing<br><br>Week 21: Dynamic exercises with eyes open with semi-globe flat side down<br>-stance: walk on 10 semi-globes<br>-dribbling: walk on 10 semi-globes and power dribble high and low<br>-aberdeen: walk on 10 semi-globes and turn ball around trunk<br>-passing on the ball of the feet: walk on 10 semi-globes and chest pass					

Study	Detailed Components of Warmup Interventions	Duration of sessions	Frequency of sessions	Duration of intervention period	Adaptation over time	Non-warmup components of the intervention and/or special equipment
	Week 22: Dynamic exercises with eyes open with semi-globe flat side down<br>-stance: walk on 10 semi-globes + shot<br>-dribbling: walk on 10 semi-globes and power dribble high and low + shot<br>-aberdeen: walk on 10 semi-globes and turn ball around trunk + shot<br>-passing on the ball of the feet: walk on 10 semi-globes and chest pass + shot					
**Emery et al., 2007 (3)**	**Balance training:**<br>5 mins sport-specific balance training warmup component for practice sessions.<br>20 minute home exercise program using a wobble board.<br>No other details provided.	5 min at practice sessions & 20 min at home	approximately 5 times per week	1 season (18 weeks)	Progression of the at home program at 2 and 4 weeks	16 inch diameter wobble board for their use at home.<br><br>Each team assigned a student team manager and a team therapist. Team therapist attended 1 session per week. Team manager attended every team session and recorded participation on an exposure sheet.
**Eils et al., 2010 (4)**	**Balance training:**<br>Six-station rotation, performed twice, with each station lasting 45 secs followed by 30 sec break and transition to next station.<br>1) Walking slowly back and forth on a balance beam, with contralateral leg swinging through and nearly touching the ground.<br>- adapt by walking faster<br>- adapt by holding a stance on the beam and using the contralateral leg to move a basketball in circles on the ground.<br>2) Single leg stance on exercise mat with other leg flexed. Lower and raise the body.<br>- adapt by working opposite a partner and passing a basketball back and forth with controlled position<br>- adapt by balancing a ball on the dorsum of the elevated foot<br>3) jump from one leg to the other on an exercise mat and control landing for 4 secs, raising the contralateral leg<br>- adapt by working with a partner and disturbing each other during the flight phase<br>- adapt by working on a soft mat<br>4) Walk up and down an inclined surface and dribble a ball<br>- adapt by adding an elastic strap around the knees and focusing on wide steps<br>- adapt by passing a ball with a partner working opposite and doing the same<br>5) maintain balance in single-leg stance, elevating contralateral leg against a resistance band<br>- adapt by doing with eyes closed<br>- adapt by moving the contralateral leg sideways and everting the lateral edge of the contralateral foot<br>6) maintain balance in single-leg stance on a tilt board. Contralateral leg is rested on an inclined surface without being loaded<br>- adapt by passing a ball with a partner<br>- adapt by elevating the contralateral leg	20 min	1 time per week	1 season - mean of 55 sessions per player	Program was intensified twice during the season by minor modifications to each station.	Balance training program used balance beams, tilt boards, and inclined surfaces.<br><br>Study team contacted coaches on a regular basis to encourage and motivate. Physiotherapist provided on-site instruction three times during the intervention period. Coach was given a printed manual that included background information on posture and proprioception.

Study	Detailed Components of Warmup Interventions	Duration of sessions	Frequency of sessions	Duration of intervention period	Adaptation over time	Non-warmup components of the intervention and/or special equipment
**Riva et al., 2016 (5)**	**Balance training:**<br>1st biennium:<br>-classic proprioceptive exercises using rocking boards and unstable surfaces<br><br>2nd biennium (sessions consisted of a sequence of repetitions lasting 30 seconds, alternating left and right limbs with a 15 second recovery period between repetitions):<br>-active management of high-frequency rocking instability (rolling and inclination) of the board<br>-feedback of vertical control<br>-assignment of specific tasks concerning board control and postural control<br><br>3rd biennium (sequence of trials lasting 30-60 seconds, alternating left and right limbs with recovery period between repetitions decreased progressively from 15 to 5 secs) :<br>-progressively longer repetitions (maximum duration of 60 secs) and minimized recovery time<br>-tasks for exploring ankle range of motion dynamically<br>-hyperfrequency instability<br>-athletes mainly performed 3 types of proprioceptive training sessions:<br>1) proprioceptive control: 18 +- 15 mins (density <50-80%)<br>2)enduring proprioceptive control and structural resilience: 25 +- 5 mins (density >=85%)<br>3) proprioceptive activation (pre-match and pre-training: 8-12 mins (density 70-80%)	1st biennium = 15-25 mins<br><br>2nd biennium =<br>• 10-20 mins 2006-2007<br>• 20-30 mins 2007-2008<br><br>3rd biennium =<br>• 15-20-25 mins 2008-2009,<br>• 12-18-24 mins 2009-2010	1st biennium = 2-3 sessions per week<br>2nd/3rd biennium = 2-4 sessions per week	6 years (3 2-year periods)	1st biennium = classic proprioceptive training using rocking boards and unstable surfaces, 2nd biennium =high frequency instability using electronic postural proprioceptive stations, 3rd biennium = hyperfrequency and high-density	Electronic proprioceptive stations
**Hewett et al., 1999 (6)**	**Static Stretching:**<br>15 minutes before Plyometrics: 3 sets, 30 secs each of calf, soleus, quadriceps, hamstring, hip flexors, IT band/low back, posterior deltoids, latissimus dorsi, pectorals, biceps<br><br>**Dynamic warmup:**<br>Before jumping exercise: skipping (2 laps), side shuffle (2 laps)<br><br>**Jumping/Plyometrics:**<br>-Wall jumps: 20 secs week 1, 25 secs week 2, 30 secs weeks 3-6<br>-Tuck jumps: 20 secs week 1, 25 secs week 2, 30 secs weeks 3-4<br>-Broad jumps hold landing: 5 reps week 1, 10 reps week 2<br>-Squat jumps: 10 secs week 1, 15 secs week 2, 20 secs weeks 3-4, 25 secs weeks 5-6<br>-Double-legged cone jumps: 30 sec/30 sec week 1 and 3, 30 sec/30 sec side to side and back to front weeks 2 and 4<br>-180-degree jumps: 20 secs week 1, 25 secs week 2<br>-Bounding in place: 20 secs week 1, 25 secs week 2<br>-Jump, jump, jump, vertical jump: 5 reps week 3, 8 reps week 4<br>-Bounding for distance: 1 run week 3, 2 runs week 4<br>-Scissors jump: 30 secs weeks 3-4<br>-Hop, hop hold landing: 5 reps/leg weeks 3-6<br>-Step, jump up, down, vertical: 5 reps week 5, 10 reps week 6<br>-Mattress jumps: 30 sec/30 sec weeks 5, 30 sec/30 sec side to side and back to front week 6<br>-Single-legged jumps distance: 5 reps/leg weeks 5-6	60-90 mins	3 days/week on alternating days	6 weeks	Increasing intensity by week	Coaches and trainers were instructed in the implementation of the program using the video tape and manual documenting all phases of training

Study	Detailed Components of Warmup Interventions	Duration of sessions	Frequency of sessions	Duration of intervention period	Adaptation over time	Non-warmup components of the intervention and/or special equipment
	-Jump into bounding: 3 runs week 5, 4 runs week 6<br><br>**Strength**:<br>Immediately after Plyometrics: 1 set each of:<br>-Pullover, bench press, Latissimus dorsi pulldown, forearm curl (12 reps)<br>-Abdominal curl, back hyperextension, leg press, calf raise, warm-down (15 reps)<br><br>Post-training: cool down walk (2 minutes), stretching (5 mins)					
**Pfeiffer et al., 2006 (7)**	"Knee Ligament Injury Prevention (KLIP) Program"<br><br>**Dynamic warmup:**<br>Stop-and-go, "W" drill, figure-eights, left/right cuts<br><br>**Jumping/Plyometrics:**<br>Phase 1: Straight jumps, tuck jumps, standing broad jumps, bound in place<br><br>Phase 2: Straight jumps, tuck jumps, 180s, double-leg jumps, single-leg lateral leaps, 45-degree lateral leaps<br><br>Phase 3: Tuck jumps, single-leg lateral leaps, single-leg forward hops, combination jumps, 180s, 45-degree lateral leaps<br><br>Phase 4: Straight jumps, single-leg forward hops, combination jumps, 180s, standing broad jump, single-leg 45 degree lateral hops	20 mins	2 times per week	2 seasons	Increasing intensity through four phases, (e.g., with two-footed drills progressing to one-footed drills, in both forward and backward directions).	Teams received personal instruction in the KLIP program, instructional videotape, and printed handouts. Coaches tracked players on their participation in weekly practices and games, and the number of KLIP training sessions per week. Schools were selected for the treatment group based on their willingness to use the KLIP program.
**LaBella et al., 2011 (8)**	**Dynamic warmup:**<br>Flexibility:<br>- Forward and backward arm swings, 20 reps each side<br>- Trunk rotations, 10 in each direction<br>- Leg swings: 10 reps front to back, 10 reps side to side<br>Running:<br>- 2 laps around court or 1 lap around field<br>- Traveling exercises, 2 lengths of court each: jogging, skipping, carioca/grapevine, side shuffle with arm swing, sprint at 100% or 75% of max, high-knee skipping, high-knee carioca, backward jog, bear crawl, butt-kickers, backward jog half-length then turn and sprint, diagonal skipping<br>Agility runs:<br>- Shuttle run between 2 rows of 5 cones, rows 50 ft apart, sprint to cone and backward jog to next cone (10 reps)<br>- Diagonal run between 2 rows of 5 cones, rows 50 ft apart, sprint to cone, turn and sprint to next cone (10 reps)<br>- Lateral shuffle between 2 rows of 5 cones, rows 15 ft apart, side shuffle from cone to cone (10 reps)	20 mins before team practices; abbreviated version (dynamic motion exercises only) before games	Frequency of practice varied depending on competitive division. Teams practiced a mean (SD) of 3.3 (1.5) times per week for a mean (SD) of 13.0 (2.5) weeks	1 season	Increasing intensity by week	Coaches received DVD with narrated videos of exercises, a laminated card listing order and frequency of exercises for use on court or field, printed educational materials about knee injury risk factors and neuromuscular exercises

Study	Detailed Components of Warmup Interventions	Duration of sessions	Frequency of sessions	Duration of intervention period	Adaptation over time	Non-warmup components of the intervention and/or special equipment
	**Jumping/Plyometrics:**<br>- Ankle bounces (10 secs week 1, 20 secs week 2, 30 secs week 3+)<br>- Tuck jumps (10 secs week 1, 20 secs week 2, 30 secs week 3)<br>- Jump in place, rotated 180 degrees (10 secs week 1)<br>- Squat jumps (10 secs week 1, 20 secs week 2, 30 secs week 3+)<br>- Broad jumps, hold landing (5 reps week 1)<br>- Jump over 3-inch cones, front to back and side to side (10 secs week 1, 20 secs week 2, 30 secs week 3+)<br>- Bounding in place (10 secs week 1)<br>- Scissor jumps (20 secs week 2)<br>- Side-to-side bounding (20 secs week 2, 30 secs weeks 4+)<br>- Single leg hop, hop stick landing (20 secs week 2, 30 secs week 3+)<br>- Jump, jump, jump, vertical jump (20 secs week 2)<br>- Single leg jump for distance, 5 reps (weeks 2-4+)<br>- Jump into bounding, 4 lengths of court (week 3)<br>- Diagonal bounding, 2 lengths of court (weeks 3-4+)<br><br>**Strength training:**<br>- Heel raises<br>- Plank and side plank<br>- Pushups<br>- Prone lifting, arms and legs together, opposite arm and leg<br>- Squats (weeks 1-3)<br>- Lunges: forward (week 1), lateral and diagonal (weeks 2-4+)<br>- Prone lifting, knees flexed to 90 degrees, heels together, hips externally rotated, lift arms/legs (weeks 2-4+)<br>- Forward and lateral walking lunges (weeks 3-4+)					
**Longo et al., 2012 (9)**	**Dynamic warmup:**<br>Running exercises, 8 minutes, along major diameter of court<br>-Straight ahead x 10<br>-Hip out x2<br>-Hip in x2<br>-circling x2<br>-circling x2<br>-Running and jumping x2<br>-quick run x2<br><br>**Strength, plyometrics, balance, 15 minutes:**<br>-Jumping: vertical 3 x 15 secs; lateral 3 times along major diameter of court; box 3 times along major diameter of court<br>-Bending with both legs 10 x 3<br>-Nordic hamstring lower 10 x 3<br>-Squats: heels raised 2 x 30 secs; walking lunges 2 x 30 secs; 1-leg squats 2 x10 (each leg)<br>-Single leg balance: holding ball 2 (each leg); throwing ball with partner 3 (each leg); testing partner 3 (each	20 mins	Teams in both groups practiced 6x per week during the first month, followed by 3-4 practices per week in subsequent months.<br><br>The IG used the FIFA11 warmup at each practice session in the first month, and at least 2x per week in the subsequent months. They also	One 9-month season	NA	coaches and team captains participated in a 1 day instruction course; coaches and each player received a poster explaining every exercise

Study	Detailed Components of Warmup Interventions	Duration of sessions	Frequency of sessions	Duration of intervention period	Adaptation over time	Non-warmup components of the intervention and/or special equipment
	leg)<br><br>**Dynamic warmup:**<br>Running exercises, 1 min and 40 sec (along major diameter of court)<br>-Running over pitch x 3<br>-Bounding run x 3<br>-Running and cutting x3		did the running exercises before match play.<br><br>The CG teams were told to use their normal warmup strategy throughout, generally 3-4x per week.			
**Aerts et al., 2013 (10)**	**Jumping/Plyometrics:**<br>- Lateral jump and hold x 8 each leg; advance to lateral jump and hold x 8 each leg PLUS lateral jump x 10 seconds<br>- Front lunge x 10; advance to walking lunge x 10; advance to lunge jump x 10 seconds<br>- squat-jump x 10<br>- Step-hold x 8 each leg<br>- Single tuck-jump, soft landing x 10 each leg; advance to double tuck jump x 8<br>- broad jump x 10 each leg<br>- Scissor jump x 8<br>- Squat jump x 10<br>- Repeated tuck jumps x 10 seconds; advance to Side-to-side tuck jump x 10 seconds;<br>- Single-legged lateral hop, hold x 8 each leg; advance to lateral hop x 10 seconds; advance to lateral hop with ball x 10 seconds<br>-Jump, single-legged hold x 8 each leg; advance to hop, hold x 8 each leg; advance to Single-legged lateral hop-hold x 5 each leg<br>- two-legged 90 degree x 8 each leg; advance to single-legged 90 degree x 8 each leg<br>- X-hop x 6 each leg<br>- Hop-hop-hold x 8 each leg; advance to crossover hop-hop-hold x 8 each leg; advance to single-legged 4-way hop-hold ball on mattress x 4 each leg<br>- Single-legged 90 degree on mattress x 8 each leg; advance to single-legged 90 degree ball x 8 each leg; advance to single-legged 180 degree x 10 each leg<br><br>**Strength training:**<br>- Cocontraction x 10 each leg<br>- Wall squat x 10; advance to squat x 10<br>- Core stability x 15<br>- Pelvic bridge x 10; advance to Pelvic bridge, single legged x 10; advance to Single-legged pelvic bridge on mattress x 10 each leg; advance to Single-legged pelvic bridge ball x 10 each leg<br>- Prone bridge (elbow-knee) hip extension, shoulder flexion x 10 each leg; advance to Prone bridge hip extension x 10 each leg; advance to Prone bridge hip extension, opposed shoulder flexion on mattress x 10 each leg	5-10 min during regular warmup	2x per week	3 months of intervention	Increasing difficulty week by week and month by month	Personalized visit by a sports physiotherapist who was part of the research team to each coach, to inform them about the intervention. Coaches were given a CD with specific information including pictures, videos, and coach's instructions on who to perform the exercises. teams also received a poster illustrating the exercises and a handout with written instructions.<br><br>Used existing gym equipment such as benches and balls.<br><br>Researchers observed each session to document whether the program was being executed. They did not interfere with the intervention but did answer questions if asked.

Study	Detailed Components of Warmup Interventions	Duration of sessions	Frequency of sessions	Duration of intervention period	Adaptation over time	Non-warmup components of the intervention and/or special equipment
**Bonato et al., 2018 (11)**	**Dynamic warmup:**<br>General activation (with ball):<br>-Jog line to line (4 basketball courts)<br>-Shuttle run (4 basketball courts)<br>-Lateral and backward running (4 basketball courts)<br>Mobility Exercises/Active Stretching:<br>-Leg swing front to back side to side 1/2 x12<br>-Lateral squats 1/2 x 12<br>-Lunge superior stretch (one basketball court)<br>-Walking quad stretch (one basketball court)<br>-Monster walks (one basketball court)<br>-Inverted hamstring stretch (one basketball court)<br>-Lateral crossover step (one basketball court)<br><br>**Strength training:**<br>-Multidirectional lunges 2/3 x 10 bilaterally<br>-Nordic hamstrings 2/3 x 5<br>-Single toe raises 2/3 x 12 bilaterally<br>-Lateral bridge 2/3 x 30 s bilaterally<br><br>**Jumping/Plyometrics:**<br>-Vertical jumps 1/2 x 12<br>-Lateral hops 2/3 x 20 bilaterally<br>-Single-legged hops 2/3 x 20 bilaterally<br>-Forward hops 2/3 x 10<br><br>**Dynamic warmup:**<br>Agility Exercises (with ball)<br>-Four-way closeout (4 basketball courts)<br>-Line drills and sprint (4 basketball courts)<br>-Zigzag cones (4 basketball courts)<br>-Four corners (4 basketball courts)<br>-Pass-sprint and layup (4 basketball courts)	30 min	4x/week before every training session during the warmup immediately before regular training	1 season	NA	NA
**Foss et al., 2018 (12)**	**Strength training:**<br>Single legged Romanian dead lift 1x each leg<br>Front lunges 10x each leg<br>Bosu lateral crunch 10 each leg<br>Box double crunch 2 x 15 reps<br>Bosu swimmers 2x 10 reps<br>Bosu double knee hold 2x 20 secs<br>Bosu flat double legged pelvic bridges 2x 10 reps<br>Swiss ball back hyperextensions 2 x 15 reps	20-25 min during pre-season; 10-15 min during competitive season.	3 times per week in pre-season; 2 times per week in competitive season	Full pre-season and season	More intensive (more minutes, more frequent) in the pre-season than during competition. Progressive difficulty of exercises	NA

Study	Detailed Components of Warmup Interventions	Duration of sessions	Frequency of sessions	Duration of intervention period	Adaptation over time	Non-warmup components of the intervention and/or special equipment
	**Jumping/Plyometrics:**<br>Lateral jump and hold 8x each leg<br>Step hold 8x each leg<br>Single-legged lateral Airex hop-hold 4x each leg<br>Single tuck jump with soft landing 2x 10 reps<br>Lunge jumps 10s rep on each leg<br>Single legged 90 degree hop hold 8x each leg<br>Unanticipated hop to stabilization 3x 5s reps on each leg<br>Hop to stabilization and reach 3x each leg				throughout the program, but detail of progression is not fully described.	
**Omi et al., 2018 (13)**	**Jumping/Plyometrics**:<br>- Rebound jump (ball catch) x10 in stage 1; Rebound jump with push (ball catch) x10 in stages 2 and 3<br>- 180 degree turn x10 in stage 1; 180 degree turn (ball catch) x10 in stages 2 and 3<br>- Front-back jump (both legs) x10 in stage 1; Front-back jump (single leg) x10 in stages 2 and 3<br>- Side jump (both legs) x10 in stage 1; Side jump (single leg) x10 in stages 2 and 3<br>- Contact jump x10 in stage 3<br>- 90 degree turn with push (ball catch) x10 in stage 3<br>- Single-leg hop (ball catch) x10 in stage 3<br>- Side jump (ball catch) x10 in stage 3<br><br>**Strength training:**<br>- Single leg squat 10x2 in stage 1; Single leg squat with dumbbell 10x2 in stages 2 and 3<br>- Side bridge 30 secs x2 in stage 1; Side bridge and hip abduction 20x2 in stage 2; Side bridge and hip abduction with moderate or strong level of the flexible band 20x2 in stage 3<br>- Hip lift (both legs) 10x2 in stage 1; Hip lift (single leg) 10x2 in stage 2; Hip lift (single leg) on Bosu ball 10x2 in stage 3<br>- Russian hamstring 10x2 in stages 1 and 2; Russian hamstring with dumbbell 10x2 in stage 3<br>- Standing hip abduction 20x2 in stage 1; Standing hip abduction 20x2, with weak level of the flexible band in stage 2 and moderate or strong level of the flexible band in stage 3<br>- Hip external rotation with the flexible band 20x2, weak resistance in stage 1, moderate resistance in stage 2, strong resistance in stage 3<br>- Sidestep walk with moderate or strong level of the flexible band 15mx2 in stage 3<br><br>**Balance training (bosu ball):**<br>- Double-leg balance and pass 30 secs x2 in stage 1; Single-leg balance and dribble 30 secs x2 in stages 2 and 3<br>- Single-leg balance 30 secs x2 in stage 1; Forward lunge on Bosu ball 10x2 in stage 2; Single leg squat on Bosu ball 10x2 in stage 3<br>- Bilateral squat on Bosu ball 20x2 in stage 1; Single leg squat on Bosu ball 20x2 in stage 2; Cross-leg hop forward on Bosu ball 20x2 in stage 3<br>- Side-hop on Bosu ball 30 secs x2 in stage 3	20 min	3 times per week	Throughout season x 8 years	Progressive difficulty, upgraded 3 times during each season	Educational sessions completed 3 times per season before each progression (stage 1, 2, 3) and 3 times during each season: common ACL mechanisms of injury; ideal trunk position and knee alignment during basketball movements. Attended by players, coaches and student athletic trainers. Followed by demonstrations of each HIP program exercise by physical therapists.<br><br>Bosu ball, band

Table A3. **GRADE Evidence Rating for Studies of Balance-Based Warm-up Interventions for Basketball Players**

Outcome (Total N)	Design<br># Studies	Risk of Bias	Consistency	Directness	Precision	Publication Bias	Overall Quality
**All Lower-Extremity Injuries**<br><br>**INT: 518**<br>**CTRL: 450**	1 cluster RCT, 1 pre-post prospective cohort study	Non-comparative design in 1 study	No serious inconsistency	No serious indirectness	Imprecision<br>Small sample in 1 study; no significant difference in larger study	Unlikely	Low
**Ankle Injuries**<br><br>**INT: 765**<br>**CTRL: 690**	3 cluster RCTs, 1 non-randomized controlled study; 1 pre-post prospective cohort study	Non-comparative design in 1 study, groups not assigned randomly in 1 comparative study	No serious inconsistency	No serious indirectness	Imprecision<br>Small numbers of ankle injuries in 4 studies	Unlikely	Low
**All Knee Injuries**<br><br>**INT: 24**<br>**CTRL: 24**	1 pre-post prospective cohort study	Non-comparative design	No serious inconsistency	No serious indirectness	Imprecision<br>Small sample sizes, very few injuries during study period, difference not significant	Unlikely	Low

Table A4. **GRADE Evidence Rating for Studies of Multicomponent Warmup Interventions for Basketball Players**

Outcome (Total N)	Design<br># Studies	Risk of Bias	Consistency	Directness	Precision	Publication Bias	Overall Quality
**All Lower-Extremity Injuries**<br><br>**INT: 657**<br>**CTRL: 511**	5 cluster RCTs	In 2 studies, intervention and control groups were not equivalent at baseline	No serious inconsistency	No serious indirectness	No serious imprecision	Unlikely	Moderate
**Ankle Injuries**<br><br>**INT: 528**<br>**CTRL: 397**	4 cluster RCTs	In 2 studies, intervention and control groups were not equivalent at baseline	No serious inconsistency	No serious indirectness	Imprecision<br>Small numbers of ankle injuries, no statistically significant differences	Unlikely	Low
**ACL Injuries**<br><br>**INT: 809**<br>**CTRL: 891**	1 cluster RCT, 2 non-randomized controlled studies, 1 pre-post prospective cohort	Non-comparative design in 1 study, groups not assigned randomly in 2 comparative studies	No serious inconsistency	No serious indirectness	Imprecision<br>Small numbers of ACL injuries, statistically significant differences in only 2 studies	Unlikely	Low
**All Knee Injuries**<br><br>**INT: 528**<br>**CTRL: 397**	4 cluster RCTs	In 2 studies, intervention and control groups were not equivalent at baseline	No serious inconsistency	No serious indirectness	Imprecision<br>Small numbers of knee injuries, statistically significant differences in only 1 study	Unlikely	Low

References

1. McGuine TA, Keene JS. The effect of a balance training program on the risk of ankle sprains in high school athletes. The American journal of sports medicine. 2006;34(7):1103-11.
2. Cumps E, Verhagen E, Meeusen R. Efficacy of a sports specific balance training programme on the incidence of ankle sprains in basketball. Journal of sports science & medicine. 2007;6(2):212-9.
3. Emery CA, Rose MS, McAllister JR, Meeuwisse WH. A prevention strategy to reduce the incidence of injury in high school basketball: a cluster randomized controlled trial. Clinical journal of sport medicine : official journal of the Canadian Academy of Sport Medicine. 2007;17(1):17-24.
4. Eils E, Schroter R, Schroder M, Gerss J, Rosenbaum D. Multistation proprioceptive exercise program prevents ankle injuries in basketball. Medicine and science in sports and exercise. 2010;42(11):2098-105.
5. Riva D, Bianchi R, Rocca F, Mamo C. Proprioceptive Training and Injury Prevention in a Professional Men's Basketball Team: A Six-Year Prospective Study. Journal of strength and conditioning research. 2016;30(2):461-75.
6. Hewett TE, Lindenfeld TN, Riccobene JV, Noyes FR. The effect of neuromuscular training on the incidence of knee injury in female athletes. A prospective study. The American journal of sports medicine. 1999;27(6):699-706.
7. Pfeiffer RP, Shea KG, Roberts D, Grandstrand S, Bond L. Lack of effect of a knee ligament injury prevention program on the incidence of noncontact anterior cruciate ligament injury. The Journal of bone and joint surgery American volume. 2006;88(8):1769-74.
8. LaBella CR, Huxford MR, Grissom J, Kim KY, Peng J, Christoffel KK. Effect of neuromuscular warm-up on injuries in female soccer and basketball athletes in urban public high schools: cluster randomized controlled trial. Archives of pediatrics & adolescent medicine. 2011;165(11):1033-40.
9. Longo UG, Loppini M, Berton A, Marinozzi A, Maffulli N, Denaro V. The FIFA 11+ program is effective in preventing injuries in elite male basketball players: a cluster randomized controlled trial. The American journal of sports medicine. 2012;40(5):996-1005.
10. Aerts I, Cumps E, Verhagen E, Mathieu N, Van Schuerbeeck S, Meeusen R. A 3-month jump-landing training program: a feasibility study using the RE-AIM framework. Journal of athletic training. 2013;48(3):296-305.
11. Bonato M, Benis R, La Torre A. Neuromuscular training reduces lower limb injuries in elite female basketball players. A cluster randomized controlled trial. Scandinavian journal of medicine & science in sports. 2018;28(4):1451-60.
12. Foss KDB, Thomas S, Khoury JC, Myer GD, Hewett TE. A School-Based Neuromuscular Training Program and Sport-Related Injury Incidence: A Prospective Randomized Controlled Clinical Trial. Journal of athletic training. 2018;53(1):20-8.
13. Omi Y, Sugimoto D, Kuriyama S, Kurihara T, Miyamoto K, Yun S, et al. Effect of Hip-Focused Injury Prevention Training for Anterior Cruciate Ligament Injury Reduction in Female Basketball Players: A 12-Year Prospective Intervention Study. The American journal of sports medicine. 2018;46(4):852-61.

Declarations

Ethics Approval and consent to participate

Not applicable

Consent for publication

Not applicable

Availability of Data and Materials

Not applicable

Competing Interests

Anna Davis, Nicholas Emptage, Dana Pounds, Donna Woo, Robert Sallis, Manuel Romero and Adam Sharp declare that they have no competing interests.

Funding

This research study was supported by Kaiser Permanente through its partnership with the National Basketball Association (NBA). The views and opinions expressed in this manuscript are the responsibility of the authors and do not represent the official views of Kaiser Permanente or the NBA.

Authors' Contributions

All authors have contributed to this manuscript in accordance with ICMJE guidelines for authorship. All authors contributed to the study conception and design. Material preparation, data collection and analysis were performed by Anna Davis, Nicholas Emptage, Dana Pounds, Donna Woo, and Manuel Romero. The first draft of the manuscript was written by Anna Davis, Nicholas Emptage, and Adam Sharp. All authors commented on draft versions of the manuscript. All authors read and approved the final manuscript.

Acknowledgments

The authors gratefully acknowledge Corrine Munoz-Plaza and Stacy Park for their contributions to this work as engaged members of our research team. We also wish to thank the members of the expert advisory committee overseeing this study for their support and advisement.
